# Evaluating the Governing Factors of Variability in Nocturnal Boundary Layer Height Based on Elastic Lidar in Wuhan

**DOI:** 10.3390/ijerph13111071

**Published:** 2016-11-01

**Authors:** Wei Wang, Feiyue Mao, Wei Gong, Zengxin Pan, Lin Du

**Affiliations:** 1 State Key Laboratory of Information Engineering in Surveying, Mapping and Remote Sensing (LIESMARS), Wuhan University, Wuhan 430079, China; wangweicn@whu.edu.cn (W.W.); weigong@whu.edu.cn (W.G.); pzx@whu.edu.cn (Z.P.); linyufocus@foxmail.com (L.D.); 2 School of Remote Sensing and Information Engineering, Wuhan University, Wuhan 430079, China; 3 Collaborative Innovation Center for Geospatial Technology, Wuhan 430079, China; 4 Hubei Collaborative Innovation Center for High-Efficiency Utilization of Solar Energy, Wuhan 430068, China; 5 School of Physics and Technology, Wuhan University, 299, Bayi Road, Wuhan 430071, China

**Keywords:** LiDAR, atmosphere, boundary layer, sensible heat flux, particle matter

## Abstract

The atmospheric boundary layer (ABL), an atmospheric region near the Earth’s surface, is affected by surface forcing and is important for studying air quality, climate, and weather forecasts. In this study, long-term urban nocturnal boundary layers (NBLs) were estimated by an elastic backscatter light detection and ranging (LiDAR) with various methods in Wuhan (30.5° N, 114.4° E), a city in Central China. This study aims to explore two ABL research topics: (1) the relationship between NBL height (NBLH) and near-surface parameters (e.g., sensible heat flux, temperature, wind speed, and relative humidity) to elucidate meteorological processes governing NBL variability; and (2) the influence of NBLH variations in surface particulate matter (PM) in Wuhan. We analyzed the nocturnal ABL-dilution/ABL-accumulation effect on surface particle concentration by using a typical case. A long-term analysis was then performed from 5 December 2012–17 June 2016. Results reveal that the seasonal averages of nocturnal (from 20:00 to 05:00 next day, Chinese standard time) NBLHs are 386 ± 161 m in spring, 473 ± 154 m in summer, 383 ± 137 m in autumn, and 309 ± 94 m in winter. The seasonal variations in NBLH, AOD, and PM_2.5_ display a deep (shallow) seasonal mean NBL, consistent with a small (larger) seasonal mean PM_2.5_ near the surface. Seasonal variability of NBLH is partly linearly correlated with sensible heat flux at the surface (R = 0.72). Linear regression analyses between NBLH and other parameters show the following: (1) the positive correlation (R = 0.68) between NBLH and surface temperature indicates high (low) NBLH corresponding to warm (cool) conditions; (2) the slight positive correlation (R = 0.52) between NBLH and surface relative humidity in Wuhan; and (3) the weak positive correlation (R = 0.38) between NBLH and wind speed inside the NBL may imply that the latter is not an important direct driver that governs the seasonal variability of NBLH.

## 1. Introduction

The atmospheric boundary layer (ABL), an atmospheric region near the Earth’s surface, is affected by surface forcing, ranging from 100 m to a few kilometers and plays an important role in the exchange of matter and energy between the surface and the free atmosphere [1]. The top of the boundary layer exhibits a blocking effect on surface pollutant dispersion, resulting in high concentrations of near-surface contaminants [2,3,4]. Therefore, an accurate study of the ABL height on diurnal, seasonal, and inter-annual timescales is significant for the study of air quality, climate, and weather forecasts [5,6]. However, the ABL structure is affected by topography [7], surface, season, day, night, and weather [8]. Moreover, the nocturnal urban boundary layer is difficult to measure because the urban heat island delays surface cooling [9,10].

Several tools, such as radiosonde (RS) [8,11], microwave radiometer [12,13], and light detection and ranging (LiDAR) [14,15,16], are applied to estimate the ABL height (ABLH). RS can accurately measure the profiles of moisture and temperature. However, sounding balloons are costly and typically launched only twice a day [17]. A microwave radiometer can also obtain the profiles of moisture and temperature, but has a low vertical resolution. An elastic backscatter LiDAR exhibits high spatial and temporal resolution and is widely used in research of atmospheric aerosol, air pollutant, atmospheric composition, and cloud [17,18,19,20]. Continuous elastic backscatter LiDAR measurements with high time resolution are employed to characterize the ABLH.

Numerous papers reported LiDAR observations of processes in the ABLH [6,14,21]. Yan et al. [22] shows that mixed layer depth is driven by the convective air motions and strongly correlated with the aerosol diurnal changes which tend to fall during the night and rise during the day by using an eye-safe micro-pulsed Mie-scattering LiDAR system. The relationships between ABLH and air pollution and between ABLH and weather conditions in China have been the focus of studies in the past decade [23,24]. Kong and Yi [25] suggest that the seasonal behavior of the surface fine particle concentration mainly depends on the seasonal variation in available volume (determined by the ABLH) for aerosol dispersion. The relationship between the daytime ABLH and the near-surface thermodynamic parameters has also been assessed; such a relationship determines meteorological processes governing the daytime ABLH variability [26]. However, these studies emphasize the ABL during the day. Therefore, the nocturnal boundary layer height (NBLH) should be analyzed in a particular region, especially if it is an urban zone with a large population.

In this study, an elastic backscatter LiDAR system for long-term NBLH detection is operated in Wuhan from 5 December 2012–17 June 2016. Three typical methods, namely, ideal profile fitting (Ideal), gradient (Grad), and wavelet covariance transform (WCT) methods, are applied to evaluate NBLH with vertical range-corrected signal (RCS) measured by an elastic LiDAR. The influences of near-surface parameters (e.g., sensible heat flux (SHF), temperature, wind speed, and relative humidity (RH)) on NBL variability are assessed. Moreover, we investigate the influence of NBLH variation on surface particulate matter (PM).

## 2. Experimental Site and Methods

### 2.1. Experimental Site and Meteorological Data

**Figure 1:**
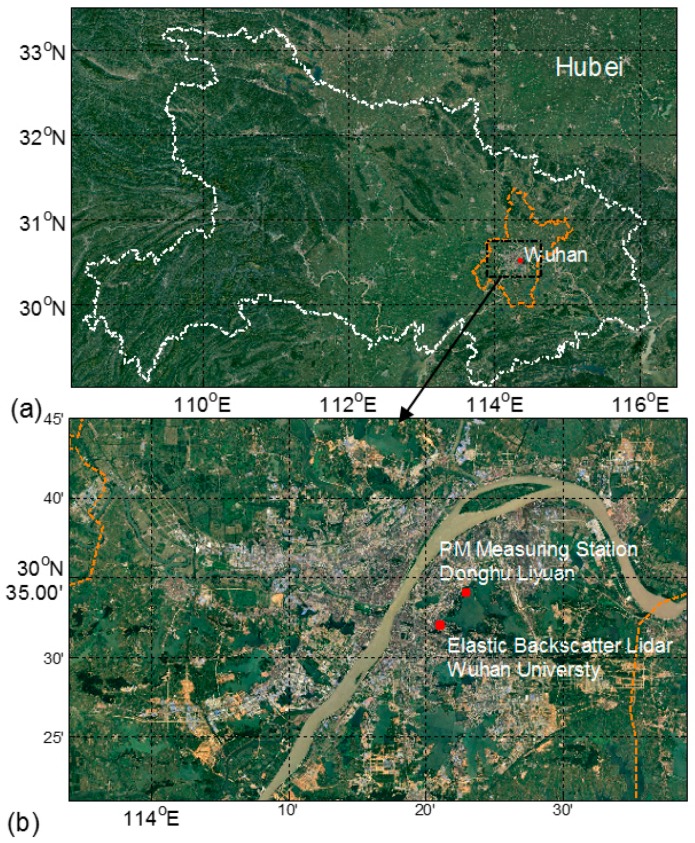
(**a**) Location of Wuhan in Hubei Province and (**b**) locations of this study in Wuhan University (ABLHs and meteorological conditions) and Donghu Liyuan (PM_2.5_ and PM_10_).

Wuhan is the largest, densely-populated city in Central China, with a population of 10.38 million (2014). This city has an area of more than 8494 km^2^ and is one of the most rapidly developing and heavily industrialized regions in Central China. Both the Yangtze and Han Rivers traverse across the main city. Wuhan experiences a typical north subtropical humid monsoon climate, with an annual average temperature of 15.8–17.5 °C and an annual average rainfall of 1050–2000 mm [27,28]. Wuhan is located in the Jianghan Plain, which is a river alluvial plain. Most areas in Wuhan are below 50 m above the sea level. Figure 1 illustrates the location and terrain of Wuhan.

An elastic LiDAR system is operated at the top of the LIESMARS building (30°32′ N, 114°21′ E, and 30 m above sea level) of Wuhan University at Wuhan (Figure 1b). An elastic LiDAR determines the nocturnal profiles of particle backscatter coefficient, particle extinction coefficient, and cloud information [19,29,30]. The PM data used are measured from Donghu Liyuan (30°34′ N, 114°22′ E), which is about 4 km northwest from the LiDAR site. The meteorological datasets with a 1 h timestep (including temperature and RH) are derived from a shared weather website [31]. The SHF data with a 6 h timestep is obtained from the reanalysis dataset of the National Center for Environmental Prediction.

### 2.2. Methods

An elastic backscatter LiDAR exhibits high spatial and temporal resolution and is widely employed to characterize the ABLH. Aerosol vertical distribution is strongly influenced by ABL thermal structures. The aerosol loading in the mixing layer is higher than that in the upper layer, leading to a sharp gradient near the mixing layer top. Three different methods, namely, Ideal, Grad, and WCT, are applied to determine the ABLH with the RCS derived from the present elastic LiDAR observations. Calculation theory for these methods is discussed in previous studies [2,14,21]. Ideal method is used to obtain boundary layer parameters by fitting elastic LiDAR-measured RCS and deriving the ABLH [32]. The Grad method determines the ABLH as the gradient maximum of the elastic LiDAR signal profile [3,6,33]. The WCT method analyzes the vertical gradients of aerosols and the rapid temporal changes in the signal time series as a function of height; this method is less affected by signal noise compared with gradient methods [14,34,35].

The overlap of the LiDAR system causes some difficulties in monitoring the shallow ABL, particularly the nocturnal boundary layer (NBL) [36]. The effect of incomplete overlap should be avoided to accurately estimate the ABLH by using a LiDAR system. The configurations of our elastic LiDAR vary at different nights because it is a self-developing LiDAR. The complete overlap heights range from 0.1–0.4 km. To ensure that the overlap minimally influences the estimation of the NBL height, we choose these cases with a complete overlap height lower than 0.15 km. Thus, the shallow NBL (below 0.15 km) cannot be determined in the present study.

Aerosol optical depth (AOD) is defined as the integral of the particle extinction coefficient along the optical path from 0–3 km [17]. The particle extinction coefficient can be retrieved by the Fernald method [37] with a fix LiDAR ratio (50 sr). We use the particle extinction coefficient at a complete overlap height (about 0.15 km) to replace the values with incomplete overlap (below 0.15 km) and, thus, avoid the overlap effects.

## 3. Analysis and Discussion

Data for analysis were obtained by elastic LiDAR at no-rain night time (i.e., 20:00–05:00 CST) from 5 December 2012–17 June 2016. The temporal resolution of the retrieved results is 15 min, and 261 days are available for the entire measured period. In this study, we only analyze the signal with obvious NBLH. Therefore, the statistical results may not completely represent the true results because of inadequate measurement data.

### 3.1. Case Analysis

**Figure 2:**
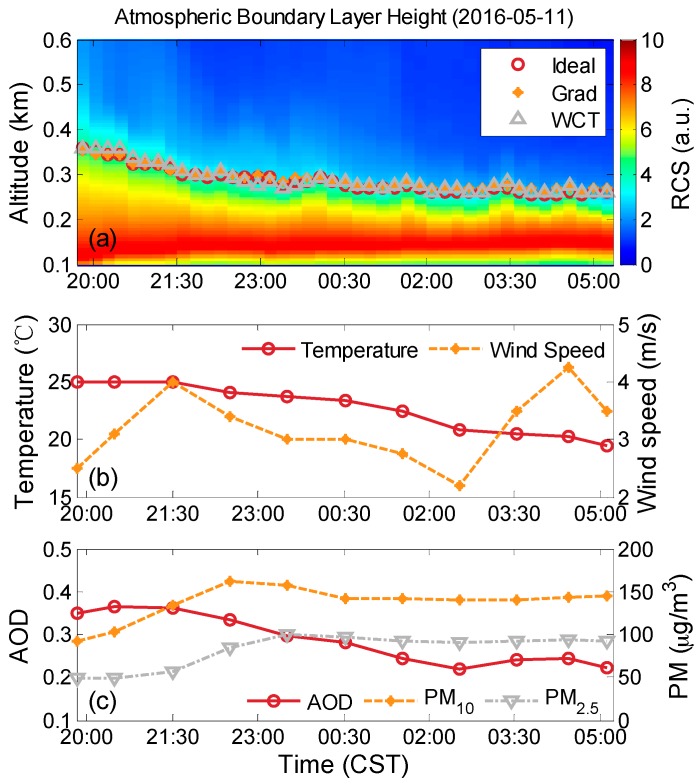
Case measured from 19:40 on 11 May 2016 to 05:20 on 12 May 2015 CST: (**a**) nocturnal boundary layer height obtained by elastic LiDAR; (**b**) temperature and wind speed; and (**c**) PM_2.5_ and PM_10_.

We selected a case observed from 19:40 (Chinese Standard Time, CST) on 11 May 2016 to 05:20 the next day. Surface temperatures vary between 20 °C and 25 °C at night. NBLH presents a slight downward trend. The variations in wind speed may minimally affect ABLH for the entire night. As shown in Figure 2c, PM_2.5_ (PM_10_) increases rapidly from 50 (90) μg/m^3^ to 100 (145) μg/m^3^ and AODs slightly decrease from 0.37 to 0.22, respectively. Thus, the declining NBLH (from 370 m to 270 m) may indicate that the ABL-dilution/ABL-accumulation effect partly contributes to the increasing PM value. At relatively high NBL, particulate near the surface can be diluted by escaping to a large volume. By contrast, the low ABLH compresses the escaping volume of particulate, resulting in increased PM_10_ and PM_2.5_ near the surface.

### 3.2. Comparison of NBLHs Obtained from Different Methods

**Figure 3:**
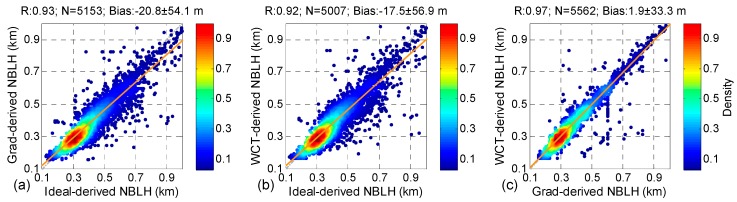
Comparison of LiDAR-derived ABLHs derived through different approaches from RCS: (**a**) the ideal profile-fitting method versus the gradient method; (**b**) the ideal profile-fitting method versus the wavelet covariance transform method; and (**c**) the gradient method versus thre wavelet covariance transform method.

Figure 3 shows the relationships of NBLHs, which were obtained through the three different methods, to RCS. The statistical parameters (i.e., correlation coefficient (R), number of comparisons (N), and bias) are plotted on top of each subfigure. R refers to the correlation coefficient between the *x*-variable and the *y*-variable. N is the valid number for comparison and defined as the range of the ABLH from 0.1 km to 1 km. The different colors in Figure 3 represent the data distribution density; red presents the maximum density, and blue shows the minimum density. Most of the ABLHs are less than 0.5 km, and the maximum density is at about 0.3 km. The high correlation coefficients (0.93, 0.92, and 0.97) and slight bias (−20.8 ± 54.1 m, −17.5 ± 56.9 m, and 1.9 ± 33.3 m) demonstrate that the results from different solutions are consistent with one another (Figure 3a–c).

### 3.3. Interannual Variability of ABLH

**Figure 4:**
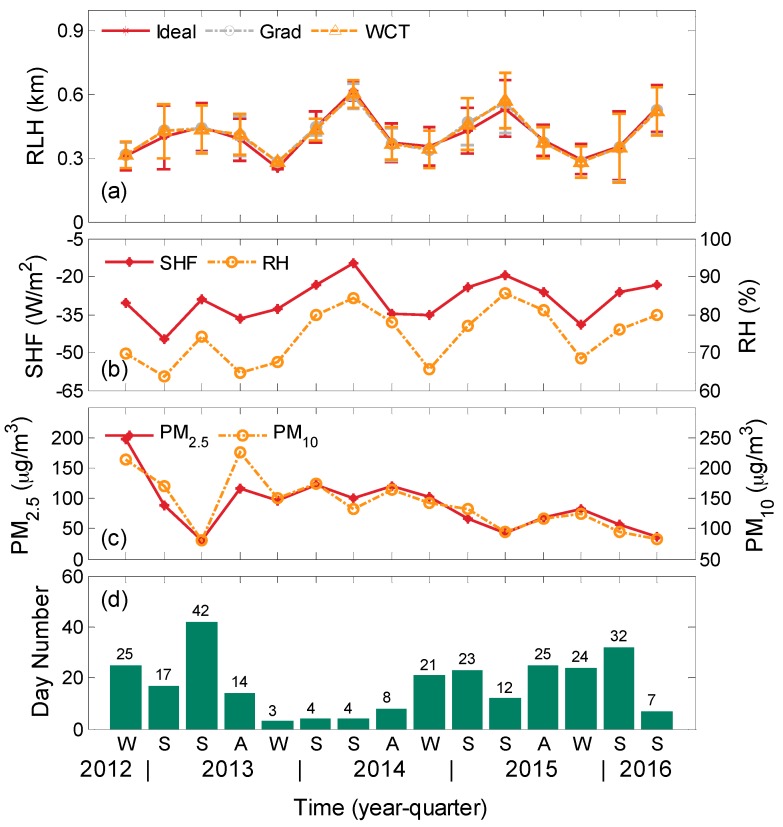
(**a**) Inter-annual quarterly mean LiDAR-derived ABLHs through three different approaches in Wuhan from 5 December 2012–17 June 2016. The error bars represent the corresponding standard deviations; (**b**) corresponding seasonal mean temperature (left *y*-axis) and wind speed (right *y*-axis) near the surface; (**c**) corresponding seasonal mean of PM_2.5_ (left *y*-axis) and PM_10_ (right *y*-axis) near the surface; and (**d**) quarterly distribution of day number in the statistical analysis.

The mean diurnal cycles for each season, March–May (MAM) (spring), June–August (JJA) (summer), September–November (SON) (autumn), and December–February (DJF) (winter), are analyzed. Figure 4a shows the quarterly mean NBLHs derived using the three different approaches with elastic LiDAR measurements in Wuhan during the entire study period. The quarterly day numbers of the available results are plotted in Figure 4d. The error bars in Figure 4a represent the corresponding standard deviations. The three sets of results are consistent with one another. A clear ABLH seasonal cycle is found through statistical analysis of long-term survey data, with a maximum mean height in summer and a minimum mean NBLH in winter.

A previous study showed that SHF and RH may significantly influence the variations in ABLH [26]. Figure 4b displays the seasonal mean SHF (left *y*-axis) and RH (right *y*-axis) near the surface for each quarter. The measured times are the same as those of the elastic LiDAR. The quarterly variations in the mean SHF and RH near the surface coincide with the variation in NBLH in each quarter [1,25]. Negative SHF at night time describes the exchange of heat from the atmosphere to the surface. The consistent variations in NBLH and SHF may indicate that the atmosphere convective motions become frequent and intense because of a slight heat exchange in summer. Therefore, the ABLHs during this season are higher than those in cold months. Some studies reported a high (low) ABLH for warmer and drier (cooler and moister) areas [26,38]. However, a reverse relationship between the quarterly variations in RH and NBLH is presented in the current study. We will explain the discrepancy in the next section.

A high (low) ABLH can diffuse (accumulate) surface aerosol particles and pollutants [25]. Figure 4c refers to the corresponding quarterly average of PM_2.5_ (left *y*-axis) and PM 10 (right *y*-axis). The trend of these PM values indicates that high pollution appears from 2012 to 2013. A negative correlation is found between PM values and NBLH. When high ABLH exists, relatively low pollution levels appear. However, the correlation is not evident, especially from 2013 to 2014. Both positive and negative correlations between ABL and fine particle concentration near the surface at day time were reported in previous studies [25,39]. Thus, the fine particle concentration near the surface generally exhibits a complex diurnal cycle.

The observed turbulent mixing parameters are weaker in 2013 than those in the other years, probably resulting in inter-annual variability. Data on 2013 are distinct from those of the other years because the former presents a high-frequency heavy pollution condition (Figure 4c), resulting in low downwelling solar radiation on the surface during the day and a high heat exchange from the atmosphere to the surface at night. These results may explain the shallower NBLH in 2013 than those in the other years.

### 3.4. Dependence of NBLH on Near-Surface Meteorological Parameters

NBLHs from the ideal method are applied in the following analysis because elastic LiDAR-derived NBLHs from the three methods are consistent. In this section, we will analyze the long-term influences of the relationship between NBLHs and near-surface meteorological parameters in Wuhan.

#### 3.4.1. Seasonal Variability

**Figure 5:**
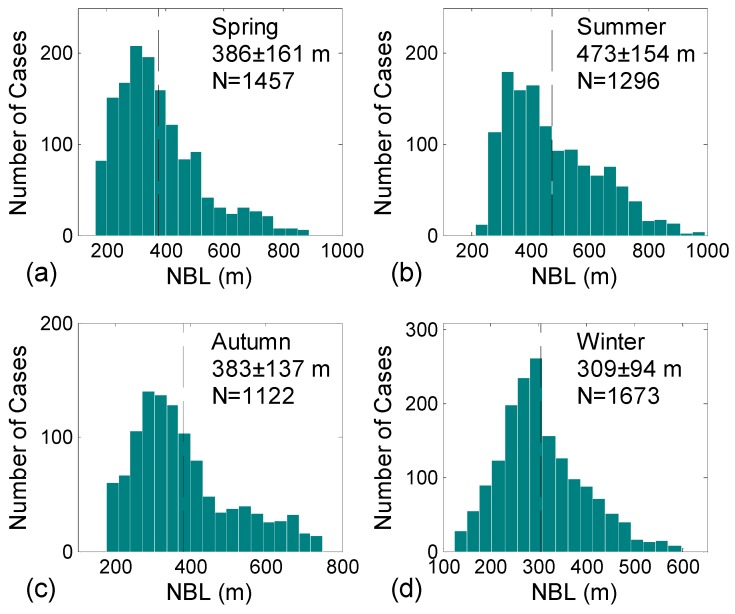
Histograms showing the frequency distribution of NBLHs for all measurements (2012–2016) as a function of seasons for bins of every 30 m interval. The dashed line on each panel marks the seasonal mean of NBLHs. Seasonal mean values with corresponding standard deviations are also shown in the top right corner of each panel. (**a**–**d**) represent spring, summer, autumn and winter, respectively.

Using frequency distribution analyses of NBLHs, we investigated the boundary layer regimes and relevant NBLH variability for the entire period. Standardized NBLH frequencies for the four seasons were considered using an NBLH bin size of 30 m (Figure 5). The summer NBLH frequency ranges from 200 m to 800 m, with the highest frequency occurring at ~350 m, which is mainly characterized with a high number of cases with deep NBLH (mean value is 473 ± 154 m). By contrast, the autumn NBLH frequency exhibits a broad and flat distribution with a wide NBLH range from 200–700 m and a mean value at 383 ± 137 m. In winter, a relatively high (low) number of cases were observed for small (large) NBLH bins. Additionally, the winter frequency distribution ranges from 150–600 m, with a mean value at 309 ± 94 m. Nevertheless, the frequency distributions yield a clear seasonal variability with the highest (lowest) mean of NBLH of 473 m (309 m) in summer (winter). The frequency distributions of NBLH differ significantly among different seasons, as indicated by the standard deviation values (Figure 5). These results are slightly higher than the recently reported measures of NBLH variability in different seasons at a rural site in France [40]. In this study, a clear seasonal dependence is found, indicating relatively higher number of cases with shallow NBLH (<200 m) in winter (67.3%) than those in summer (33.9%) and spring (54.5%). The higher results in the present study may be due to the inability to obtain the NBLH below 150 m.

**Figure 6:**
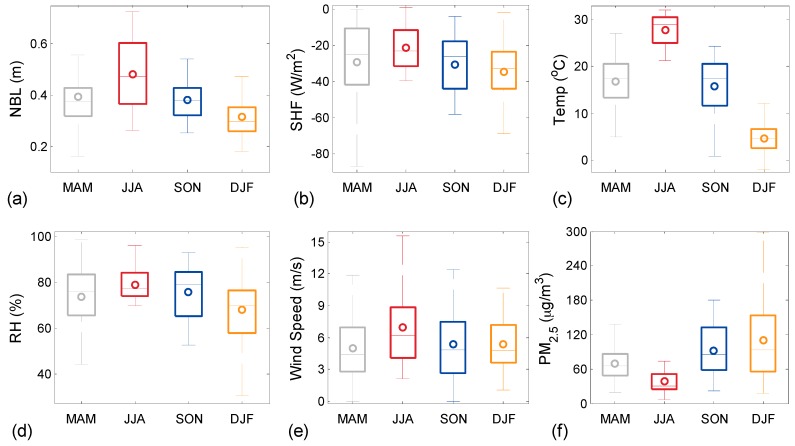
Corresponding mean seasonal variability based on the monthly-mean values for the period between December 2012 and June 2016 via box-and-whisker analyses. In each box solid line indicates the median and the extent of boxes, 25th and 75th percentiles. The central circle is the mean value, and the external short lines are the maximum and minimum. (**a**–**f**) represent seasonal variability of NBLH, SHF, surface temperature, relative humidity, wind speed and PM_2.5_, respectively.

Figure 6 presents different aspects of near-surface forcing on NBLH variability at seasonal timescales. We consider near-surface meteorological parameters including SHF (W·m^−2^), RH (%), wind speed (m·s^−1^), and temperature (°C). We performed box-and-whisker analyses to represent the seasonal variations in NBLH and these near-surface meteorological parameters. All parameters exhibit a clear seasonal variability and cyclic behavior, indicating high (low) temperature of NBLH in summer (winter) months. Additionally, the annual cycle is evident for SHF, with mean values reaching the maximum in spring and summer and the minimum during winter months. The RH parameter features a clear seasonal cycle, that is, wet condition in summer and dry condition in winter (Figure 6d) are due to high amounts of precipitation and temperature in summer than those in winter months at Wuhan. Overall, the three datasets (SHF, Temp, RH, and NBLH) show good agreement and seasonal similarities. However, RH shows an opposite annual cycle compared with those in the other studies which suggest higher (lower) ABLH in drier (moister) areas [26,38]. The inconsistent results could be due to the fact that previous studies were performed during the day, whereas the present study was conducted at night. Moreover, perhaps near-surface RH may not be a crucial factor for NBLH variation.

Using the daily 20:00 CST radiosonde-derived profiles of horizontal wind speed within the LiDAR-derived NBLH, we quantified the dependence of variability of NBLH on mean wind speed. Wind speed is an important parameter used to investigate detailed eddy structures and scaling turbulence estimating CBL height [1,26]. We estimated the average wind speed within altitudes between 50 m and the middle of the NBLH obtained with radiosonde measurements; the method is similar to that reported in a previous study [26]. A clear wind speed seasonal cycle is usually found through analysis of long-term survey data, with a maximum mean wind speed in summer and a minimum mean wind speed during winter.

We also plotted the quarterly variations in PM_2.5_ (μg/m^3^) in Figure 6f to investigate the relationship of NBLH and near-surface pollution level. The seasonal mean values of AOD at 500 nm obtained from sun photometer on 2007–2013 in Wuhan are 1.03 ± 0.52, 1.13 ± 0.71, 1.07 ± 0.75, and 1.05 ± 0.60 for spring, summer, autumn, and winter, respectively [27]. Hence, a deep (shallow) seasonal mean NBL is consistent with a small (large) seasonal mean PM_2.5_ near the surface. The opposite seasonal variations in AOD and PM_2.5_ may partly explain the nocturnal ABL-dilution/ABL-accumulation effect on surface particle concentration.

#### 3.4.2. Dependence of NBLH on Near-Surface Parameters on Seasonal Scale

**Figure 7:**
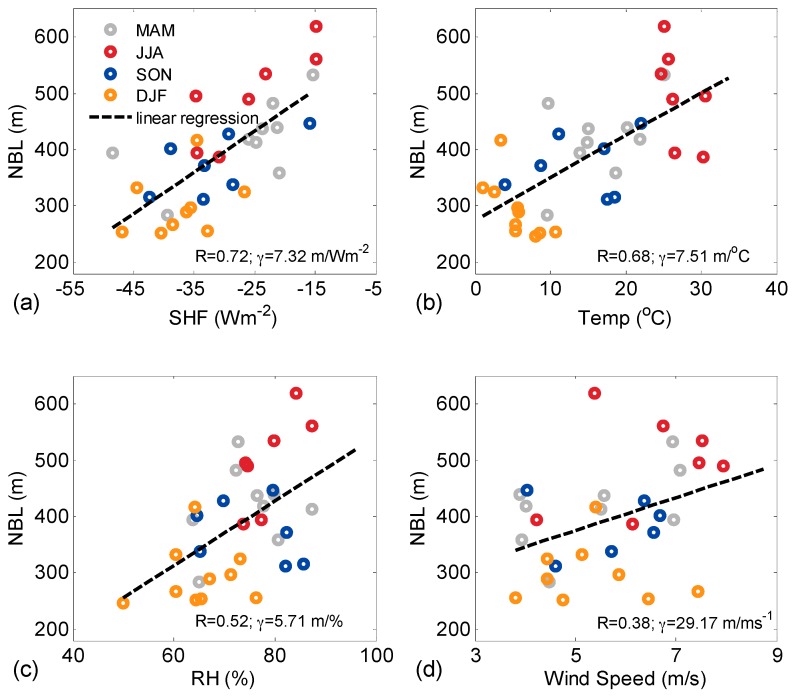
Scatterplots and regression analysis between monthly mean values of NBLH and (**a**) SHF; (**b**) temperature; (**c**) RH and (**d**) wind speed for the entire measurement period. The linear regression (dashed black line) and corresponding correlation coefficient and gradient values (γ, m/Wm^2^) are shown in each panel. The colors of the circles correspond to different seasons (color bar in Figure 7a).

The relationship between NBLH and SHF must be investigated to elucidate physical processes governing seasonal variability [26]. The scatterplots illustrate the regression analyses for the entire measured period (Figure 7a). The relationships between NBLH and the other parameters (temperature, RH and wind speed) are plotted in the same figure. The colors of the circles in each panel mark different seasons.

The relationship between NBLH and SHF (R = 0.72) is not fully linear, particularly during summer when the NBLH values are the highest; by contrast, SHF values remain in the same range among all seasons. These results are similar to the findings reported by other researchers [26]. Monthly mean nocturnal SHFs vary from −37 (winter) to −25 (summer) W/m^2^. Seasonal separation is unclear for different seasons. Autumn and winter SHFs are close (ranging from −60 to 0 W/m^2^). Spring SHF ranges from −85 W/m^2^ to 0 W/m^2^, whereas winter SHF ranges from −40 W/m^2^ to 0 W/m^2^. Thus, in spite of highest SHF in summer, the linearity between SHF and NBLH is not well developed (R = 0.72). In summer, in spite of the limited SHF, NBLH reaches higher altitudes than that in spring, which could be attributed to other sources that influence NBLH. These sources may include advection of different air masses from adjacent areas, presence of clouds, and temporal shift in the times of NBLH and SHF [26]. Yi et al. [41] discussed the relationship between SHF and ABL variability, in which ABL increases for more than 3 h even after the SHF reached the daytime peak value. Additionally, some errors in measurement of SHF and NBLH may influence the result.

The relationship between NBLH and surface temperature is shown in Figure 7b. The positive correlation (R = 0.68) indicates that high (low) NBLH corresponds warm (cool) condition. Similarly, the relationship between NBLH and surface RH exhibits a slight positive correlation (R = 0.52; Figure 7c). Other studies reported the dependence of ABLH on surface temperature and moisture. For example, Molod et al. investigated spatial variability of ABLH via NOAA wind profiler network data [38]. Pal et al. reported the dependences of ABLH on surface temperature and moisture at different time scales [21]; in this study, high (low) ABLH was observed for warm and dry (cool and moist) areas. Therefore, the relationship between NBLH and surface temperature reported in previous studies coincides with the present analysis, but the relationship between NBLH and surface RH is inconsistent. Thus, RH may play an opposite or insignificant influence on variations in NBLH in Wuhan.

SHF is an important surface-based driver of ABLH but is not the sole driver that generating day-to-day variability in ABLH. Vilà-Guerau de Arellano et al. investigated the roles of ABL wind speed in modulating ABL growth in the morning [42]. Pal et al. suggested that ABLHs are correlated with SHF under low wind speed conditions [26]. In the present analysis, the weak positive correlation (R = 0.38) between NBLH and wind speed inside NBL is shown in Figure 7d. The result implies that wind speed is not a crucial direct driver that governs the seasonal variability of NBLH.

## 4. Conclusions

We present a long-term analysis on the variability of main parameters of the NBLH diurnal cycle. A long-term dataset of NBLH was obtained by applying three detection methods on the LiDAR-derived backscatter profiles from 5 December 2012–17 June 2016. We also explored the mechanism through which other parameters may affect the variability of ABL parameters and the influence of NBLH on surface PM at seasonal and inter-annual timescales in Wuhan. Analyses were conducted using atmospheric parameters including NBLH, SHF, temperature and RH, NBL mean wind speed, and PM value. By analyzing the composites of NBLH and different meteorological parameters, we pointed out that the near-surface thermodynamics plays an integral role in governing NBLH at seasonal and inter-annual timescales. The seasonal variations of NBLH, AOD and PM_2.5_ display a deep (shallow) seasonal mean NBL, consistent with a low (high) seasonal mean PM_2.5_ near the surface. Seasonal variability of NBLH is partly linearly correlated with SHF on the surface (R = 0.72). Linear regression analyses between NBLH and other parameters show the following: (1) the positive correlation (R = 0.68) between NBLH and surface temperature indicates that high (low) NBLH corresponds to warm (cool) condition; (2) similar slight positive correlation (R = 0.52) between NBLH and surface relative humidity in Wuhan; and (3) the weak positive correlation (R = 0.38) between NBLH and wind speed inside NBL may imply that wind speed is not a crucial direct driver that governs the seasonal variability of NBLH.

Our analysis indicates that the NBL is difficult to quantitatively characterize. Future studies should determine NBLH by using several tools, such as wind LiDAR, radiosonde, and satellite-based LiDAR. The relationship between NBLH and other optical parameters or synoptic conditions requires an in-depth study.

## References

[1] Stull R.B. (1988). An Introduction to Boundary Layer Meteorology.

[2] Granados-Muñoz M., Navas-Guzmán F., Bravo-Aranda J., Guerrero-Rascado J., Lyamani H., Fernández-Gálvez J., Alados-Arboledas L. (2012). Automatic determination of the planetary boundary layer height using lidar: One-year analysis over southeastern spain. J. Geophys. Res. Atmos..

[3] Luo T., Yuan R., Wang Z. (2014). Lidar-based remote sensing of atmospheric boundary layer height over land and ocean. Atmos. Meas. Tech..

[4] Mao F., Duan M., Min Q., Gong W., Pan Z., Liu G. (2015). Investigating the impact of haze on modis cloud detection. J. Geophys. Res. Atmos..

[5] McGrath-Spangler E.L., Denning A.S. (2013). Global seasonal variations of midday planetary boundary layer depth from calipso space-borne lidar. J. Geophys. Res. Atmos..

[6] Tsaknakis G., Papayannis A., Kokkalis P., Amiridis V., Kambezidis H., Mamouri R., Georgoussis G., Avdikos G. (2011). Inter-comparison of lidar and ceilometer retrievals for aerosol and planetary boundary layer profiling over Athens, Greece. Atmos. Meas. Tech..

[7] Pal S., De Wekker S., Emmitt G. (2016). Investigation of the spatial variability of the convective boundary layer heights over an isolated mountain: Cases from the materhorn-2012 experiment. J. Appl. Meteorol. Climatol..

[8] Zhang Y., Gao Z., Li D., Li Y., Zhang N., Zhao X., Chen J. (2014). On the computation of planetary boundary-layer height using the bulk richardson number method. Geosci. Model Dev..

[9] Allegrini I., Febo A., Pasini A., Schiarini S. (1994). Monitoring of the nocturnal mixed layer by means of participate radon progeny measurement. J. Geophys. Res. Atmos..

[10] Oke T. (1995). The Heat Island of the Urban Boundary Layer: Characteristics, Causes and Effects. Wind Climate in Cities.

[11] Seidel D.J., Ao C.O., Li K. (2010). Estimating climatological planetary boundary layer heights from radiosonde observations: Comparison of methods and uncertainty analysis. J. Geophys. Res. Atmos..

[12] Pospichal B., Crewell S. (2007). Boundary layer observations in west africa using a novel microwave radiometer. Meteorol. Z..

[13] Wang Z., Cao X., Zhang L., Notholt J., Zhou B., Liu R., Zhang B. (2012). Lidar measurement of planetary boundary layer height and comparison with microwave profiling radiometer observation. Atmos. Meas. Tech..

[14] Baars H., Ansmann A., Engelmann R., Althausen D. (2008). Continuous monitoring of the boundary-layer top with lidar. Atmos. Chem. Phys..

[15] Korhonen K., Giannakaki E., Mielonen T., Pfüller A., Laakso L., Vakkari V., Baars H., Engelmann R., Beukes J., Van Zyl P. (2014). Atmospheric boundary layer top height in south africa: Measurements with lidar and radiosonde compared to three atmospheric models. Atmos. Chem. Phys..

[16] Barlow J.F., Dunbar T., Nemitz E., Wood C.R., Gallagher M., Davies F., O’Connor E., Harrison R. (2011). Boundary layer dynamics over London, UK, as observed using doppler lidar during repartee-II. Atmos. Chem. Phys..

[17] Wang W., Gong W., Mao F., Zhang J. (2015). Long-term measurement for low-tropospheric water vapor and aerosol by raman lidar in Wuhan. Atmosphere.

[18] Zhang J., Huang C., Gong W. (2014). An algorithm for retrieving the atmospheric aerosol extinction coefficient via raman lidar data. Lasers Eng..

[19] Mao F., Gong W., Logan T. (2013). Linear segmentation algorithm for detecting layer boundary with lidar. Opt. Express.

[20] Mao F.Y., Gong W., Ma Y.Y. (2012). Retrieving the aerosol lidar ratio profile by combining ground- and space-based elastic lidars. Opt. Lett..

[21] Pal S., Behrendt A., Wulfmeyer V. (2010). Elastic-backscatter-lidar-based characterization of the convective boundary layer and investigation of related statistics. Ann. Geophys..

[22] Yan Q., Hua D., Wang Y., Li S., Gao F., Wang L., Liu C., Zhang S. (2013). Observations of the boundary layer structure and aerosol properties over Xi’an using an eye-safe mie scattering lidar. J. Quant. Spectrosc. Radiat. Transf..

[23] He Q., Mao J., Chen J., Hu Y. (2006). Observational and modeling studies of urban atmospheric boundary-layer height and its evolution mechanisms. Atmos. Environ..

[24] Fan S., Wang B., Tesche M., Engelmann R., Althausen A., Liu J., Zhu W., Fan Q., Li M., Ta N. (2008). Meteorological conditions and structures of atmospheric boundary layer in October 2004 over Pearl River Delta Area. Atmos. Environ..

[25] Kong W., Yi F. (2015). Convective boundary layer evolution from lidar backscatter and its relationship with surface aerosol concentration at a location of a central China megacity. J. Geophys. Res. Atmos..

[26] Pal S., Haeffelin M. (2015). Forcing mechanisms governing diurnal, seasonal, and interannual variability in the boundary layer depths: Five years of continuous lidar observations over a suburban site near Paris. J. Geophys. Res. Atmos..

[27] Wang L., Gong W., Xia X., Zhu J., Li J., Zhu Z. (2015). Long-term observations of aerosol optical properties at wuhan, an urban site in central China. Atmos. Environ..

[28] Wang W., Gong W., Mao F., Pan Z., Liu B. (2016). Measurement and study of lidar ratio by using a raman lidar in central China. Int. J. Environ. Res. Public Health.

[29] Lifeng H., Wei G., Jun L., Feiyue M., Lianfa L. (2012). Signal splicing of dual-receiver mie scattering lidar in atmospheric remote sensing. J. Remote Sens..

[30] Mao F., Wang W., Min Q., Gong W. (2015). Approach for selecting boundary value to retrieve mie-scattering lidar data based on segmentation and two-component fitting methods. Opt. Express.

[31] Weather Underground Historical Weather. https://www.wunderground.com/history/.

[32] Steyn D.G., Baldi M., Hoff R.M. (1999). The detection of mixed layer depth and entrainment zone thickness from lidar backscatter profiles. J. Atmos. Ocean. Technol..

[33] Hayden K., Anlauf K., Hoff R., Strapp J., Bottenheim J., Wiebe H., Froude F. (1997). The vertical chemical and meteorological structure of the boundary layer in the lower fraser valley during Pacific′93. Atmos. Environ..

[34] Mao F.Y., Gong W., Song S.L., Zhu Z.M. (2013). Determination of the boundary layer top from lidar backscatter profiles using a haar wavelet method over Wuhan, China. Opt. Laser Technol..

[35] Brooks I.M. (2003). Finding boundary layer top: Application of a wavelet covariance transform to lidar backscatter profiles. J. Atmos. Ocean. Technol..

[36] Pal S. (2014). Monitoring depth of shallow atmospheric boundary layer to complement lidar measurements affected by partial overlap. Remote Sens..

[37] Fernald F.G., Herman B.M., Reagan J.A. (1972). Determination of aerosol height distributions by lidar. J. Appl. Meteorol..

[38] Molod A., Salmun H., Dempsey M. (2015). Estimating planetary boundary layer heights from noaa profiler network wind profiler data. J. Atmos. Ocean. Technol..

[39] Pal S., Lee T., Phelps S., De Wekker S. (2014). Impact of atmospheric boundary layer depth variability and wind reversal on the diurnal variability of aerosol concentration at a valley site. Sci. Total Environ..

[40] Pal S., Lopez M., Schmidt M., Ramonet M., Gibert F., Xueref-Remy I., Ciais P. (2015). Investigation of the atmospheric boundary layer depth variability and its impact on the 222 rn concentration at a rural site in France. J. Geophys. Res. Atmos..

[41] Yi C., Davis K.J., Berger B.W., Bakwin P.S. (2001). Long-term observations of the dynamics of the continental planetary boundary layer. J. Atmos. Sci..

[42] Vilà-Guerau de Arellano J., Patton E.G., Karl T., van den Dries K., Barth M.C., Orlando J.J. (2011). The role of boundary layer dynamics on the diurnal evolution of isoprene and the hydroxyl radical over tropical forests. J. Geophys. Res. Atmos..

